# Phenylketonuria screening in Iranian newborns: a systematic review and meta-analysis

**DOI:** 10.1186/s12887-020-02230-6

**Published:** 2020-07-24

**Authors:** Mehdi Shokri, Parviz Karimi, Hadis Zamanifar, Fatemeh Kazemi, Gholamreza Badfar, Milad Azami

**Affiliations:** 1 Department of Pediatrics, School of Medicine, Ilam University of Medical Sciences, Ilam, Iran; 2 School of Nursing and Midwifery, Ahvaz Jundishapour University of Medical Sciences, Ahvaz, Iran; 3 School of Medicine, Qazvin University of Medical Sciences, Qazvin, Iran; 4 Department of Pediatric, Faculty of Medicine, Ahvaz Jundishapour University of Medical Sciences, Ahvaz, Iran; 5 School of Medicine, Ilam University of Medical Sciences, Ilam, Iran

**Keywords:** Phenylketonuria, Iran, Newborn, Meta-analysis

## Abstract

**Background:**

Phenylketonuria (PKU), which is characterized by a deficiency of phenylalanine hydroxylase activity, is an autosomal recessive disorder of phenylalanine (Phe) metabolism. Newborn screening is the main population-based public health screening program that allows successful identification and treatment of PKU with low-Phe diet. The aim of this study was to evaluate the epidemiology of PKU screening in Iranian newborns.

**Methods:**

The present study was designed based on MOOSE protocol and reporting was done in accordance with the PRISMA guidelines. The protocol of this systematic review was published in PROSPERO before it was performed (CRD42020162626). A comprehensive search was done in 10/10/2019 to find related literature on international online databases Web of Science, Scopus, EMBASE, Science Direct, PubMed/Medline, EBSCO, CINAHL, Cochrane Library, national online databases and the Google Scholar search engine. Heterogeneity among studies was assessed by I^2^ index and Q test. All meta-analyses were performed using Comprehensive Meta-Analysis Software ver. 2. *P* < 0.05 was considered significant.

**Result:**

Finally, 18 studies with 3,339,327 Iranian neonates were included. The prevalence of suspected hyperphenylalaninemia **(**HPA) was estimated to be 45.6/100,000 (95% CI: 23.9–87.1). The prevalence of suspected HPA in girls and boys infants in Iran was estimated to be 38.0/100,000 (95% CI: 15.1-95.5) and 43.3/100,000 (95% CI: 16.2-116.2), respectively. The prevalence of PKU was estimated to be 16.5/100,000 (95% CI: 12.9–21.2). The prevalence of PKU in girls and boys infants was estimated to be 13.3/100,000 (95% CI: 7.5–15.8) and 10.9/100,000 (95% CI: 7.5–15.8), respectively. The prevalence of mild to moderate HPA was estimated 9.7/100,000 (95% CI: 5.1–18.4) and the prevalence of classical PKU was estimated 4.4/100,000 (95% CI: 2.5–7.8). Sensitivity analysis for all meta-analysis with the omission of one study showed that overall estimation is still robust.

**Conclusion:**

The results of this meta-analysis showed that PKU is prevalent in Iranian neonates**.** It should be considered that for PKU there is a highly effective dietary treatment which can prevent the clinical symptoms of PKU if initiated early after detection by newborn screening.

## Background

Phenylketonuria (PKU), which is characterized by a deficiency of phenylalanine hydroxylase (PAH) activity, is an autosomal recessive disorder of phenylalanine (Phe) metabolism [1]. If left untreated, high blood Phe concentrations pass through the blood brain barrier and cause harmful effects on the growth and function of brain [2].

The main treatment for PKU is low Phe diet. It is recommended that treatment be started as soon as possible and continue throughout life. Although a restricted diet is beneficial for PKU patients, long-term adherence to diet is a difficult challenge, especially for teenagers and those preparing for pregnancy [3].

If urine tyrosine and tetrahydrobiopterin levels are normal and Phe levels are above 20 mg/dl, between 10 and 20 mg/dl, and between 2 and 10 mg/dl, newborns are diagnosed with severe or classical PKU, mild PKU and hyperphenylalaninemia (HPA), respectively [4].

Nowadays, clinical manifestations of classical PKU are rarely reported in developed countries, where newborn screening (NBS) is common. NBS is the main population-based public health screening program that allows successful identification and treatment of PKU with low-Phe diet. Early dietary treatment leads to normal results for patients with this disorder [5]. The first NBS program appeared in the United States in the early 1960s [6] and then spread to most developed countries [7]. PKU can be easily detected in heel prick test 24 h after birth using novel diagnostic methods [8]. There are various methods for detecting PKU in dried blood spot (DBS) sampling, such as fluorometric and colorimetric methods [9], enzymatic method [10, 11], high-performance liquid chromatographic (HPLC) [10], and new methods such as Tandem Mass Spectrometry [12, 13].

Numerous studies have shown that the prevalence of PKU is inconsistent in different Iranian studies and is still a challenging issue [14–31]. Meta-analysis is a statistical method for combining the data of multiple studies with the same goal. When the effect size is consistent between studies, meta-analysis can be used to identify this common effect. Finally, meta-analysis results can provide a more accurate estimate of the impact of treatment or risk factors for disease or other outcomes by combining individual studies [32–34]. The aim of this study was to evaluate the epidemiology of PKU screening in Iranian newborns.

## Method

### Study protocol

The present study was designed based on Meta-analyses Of Observational Studies in Epidemiology (MOOSE) protocol [35] and reporting was done in accordance with the Preferred Reporting Items for Systematic Reviews and Meta-analysis (PRISMA) guidelines [34]. Given the type of study, the approval of the Ethics Committee was not required. All study phases were performed independently by two authors. In cases where there were disagreements, they were resolved through group discussion. The protocol of this systematic review was published in PROSPERO before it was performed (CRD42020162626). Available from: https://www.crd.york.ac.uk/prospero/display_record.php?RecordID=162626.

### Search strategy

A comprehensive search was done in 10/10/2019 to find related literature on international online databases Web of Science, Scopus, EMBASE, Science Direct, PubMed/Medline, EBSCO, CINAHL, Cochrane Library (Cochrane Database of Systematic Reviews - CDSR), and national online databases Barakat Knowledge Network System (http://health.barakatkns.com), Magiran (http://www.magiran.com/), Regional Information Center for Science and Technology (RICST) (http://en.ricest.ac.ir/), Scientific Information Database (SID) (http://www.sid.ir/), Civilica (https://www.civilica.com/), Iranian Research Institute for Information Science and Technology (IranDoc ((https://irandoc.ac.ir), Iranian National Library (http://www.nlai.ir/) and the Google Scholar search engine. The search was carried out without limitation in time and language. Keywords were: “Metabolism, Inborn Errors” [Mesh], “Metabolic Diseases” [Mesh], “Amino Acid Metabolism, Inborn Errors” [Mesh], “Phenylketonurias” [Mesh] and “Iran” [Mesh].

The keywords were combined using boolean operator “AND” and “OR”. An example of a combined search in the Pubmed database was as follows: ((((“Metabolism, Inborn Errors” [Mesh]) OR “Metabolic Diseases” [Mesh]) OR “Amino Acid Metabolism, Inborn Errors” [Mesh]) OR “Phenylketonurias” [Mesh]) AND “Iran” [Mesh].

Search keywords were regulated based on minor specifications and differences in the syntax rules of each database. Reference lists of all retrieved articles were manually reviewed to identify all potential studies.

### Inclusion and exclusion criteria

PICO (Patient, Population, or Problem; Intervention, Prognostic Factor, or Exposure; Comparison or Intervention (if appropriate); Outcome) [36] for inclusion and exclusion criteria were determined as follows: Inclusion criteria were all epidemiological studies about the prevalence of PKU that have been peer-reviewed at least in the form of abstract. Exclusion criteria were: 1) duplicate studies; 2) sample size other than infants (over 28 days of age); 3) non-random sample size; 4) non-Iranian studies; 5) being irrelevant; 6) sample size smaller than 200 participants; 7) participants with certain diseases (e.g. mental retardation and etc); 8) unknown diagnostic intervention; 9) poor quality qualitative evaluation; 10), case reports, review articles, and letters to the editor without quantitative data.

### Study selection

The title and abstract of all identified documentations were screened. Then, we evaluated the full text of the articles according to the inclusion and exclusion criteria. Finally, the raised disagreements were discussed and resolved in the presence of all authors.

### Definitions

Suspected cases of HPA were defined as Phe serum levels in primary NBS (in some sources 2 mg/dl and in some 4 mg/dl), and PKU was diagnosed in suspected cases of HPA after confirmatory tests. In this study, PKU it relates to all degrees of HPA, and PKU was classified into two categories: 1. Phe concentrations between (2 or 4) and 20 were considered as mild to moderate HPA, and 2. Phe concentrations above 20 were considered as classical PKU [4].

### Data extraction

Data extracted by the authors included first author’s name, email of the corresponding author or the first author, year of publication, region/province, year of study, sample size (total, boys and girls), data collection method, diagnostic criteria, prevalence for each variable (suspected HPA, PKU, and types of PKU), and finally the extracted data were entered into Excel software (XP professional edition; Microsoft, Redmond, Washington, USA).

For duplicate publications, we contacted the corresponding author or the first author to clarify the original publication, and if we did not receive a response, we selected the study with the largest number of participants for overlapping cases. We also contacted the corresponding author when the article data was incomplete or ambiguous and resolved the problem.

### Quality evaluation

As all studies eventually included the prevalence, so the quality of the studies was evaluated using a checklist for cross-sectional/prevalence studies by the modified Newcastle-Ottawa Scale (NOS) [37]. The quality of articles was classified into three categories of low, medium and high. Scoring was considered 0–5, 6–7, and 8–9, respectively, and studies with poor quality excluded.

### Statistical analysis

Heterogeneity among studies was assessed by I^2^ and Q tests. Interpretation of heterogeneity based on I^2^ Index is as follows: less than 25% (low heterogeneity), 25–49% (moderate heterogeneity) and 50–75% (considerable heterogeneity), and greater than 75% (high heterogeneity), and *P*-value ​​less than 0.10 is statistically significant [38, 39]. In cases of low heterogeneity, the fixed effects model was used, and in other cases, the random effects model was used for data integration. Girls-boys Odds Ratio (OR) was used to indicate the effect of gender on suspected HPA and PKU, using HPA and PKU positive cases in both genders and the total sample size. To explore the cause of heterogeneity, subgroup analysis was done based on the region and the province of study, and sensitivity analysis was used to measure the overall estimation power by omitting one study at a time. Mixed-effects meta-regression was used to investigate the association between continuous variables such as the effect of time of study on prevalence. Publication bias was assessed by a visual survey of the funnel plot for skewed distribution, and using the Begg and Egger’s tests [40, 41]. All meta-analyses were performed using Comprehensive Meta-Analysis Software (CMA) ver. 2. *P* < 0.05 was considered significant in all tests.

## Results

### Search results and the features of studies included the meta-analysis

**Fig. 1:**
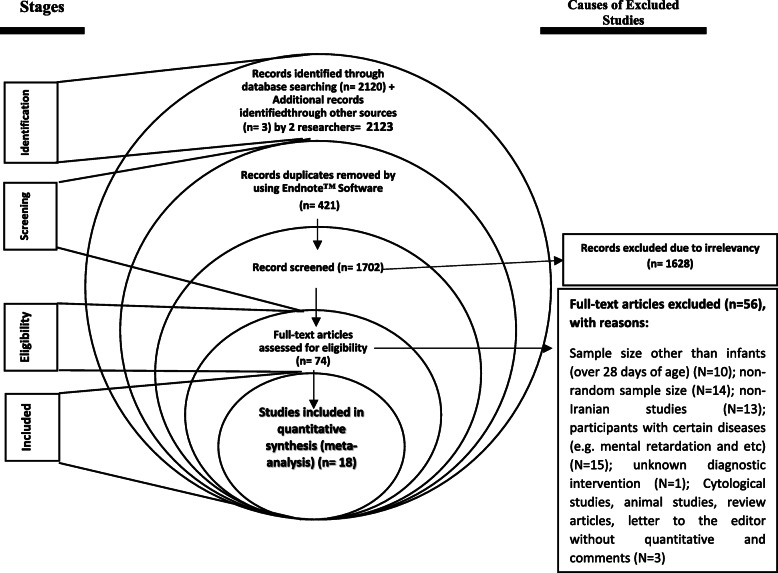
PRISMA flowchart

Figure 1 shows the flowchart of the selection of studies. Systematic search on databases and references identified 2126 related papers. Subsequently, 421 duplicate articles and 1672 unrelated articles were removed by reviewing the title and abstract. Then, 56 studies were excluded after reviewing the full-text since they did not meet the eligibility criteria. Finally, 18 studies with 3,339,327 Iranian neonates were included (Fig. 1).

Studies by Abbaskhanian A. et al. [16], Motamedi N. et al. [18], and Ganji F. et al. [28] were considered as more than one study each since they reported information in more than one population. All studies had appropriate quality to enter the meta-analysis (Table 1).

**Table:** Table 1 Summary of characteristics in studies into a meta-analysis

Ref	First author, Published Year	Year	Place	Design	Age (day)	Method	Sample size			Suspected HPA^**a**^	PKU^**b**^	Classic PKU	HPA	Quality
							**Female**	**Male**	**All**					
[14]	Senemar S, 2009	2000–5	Fars	Screening program	3	Fluorometric			70,477		15	2	13	Medium risk
[15]	Habib A, 2010	2004–7	Fars	Screening program	3–5	Colorimetric and HPLC	87,091	88,143	175,235	30	28			Medium risk
[16]	Abbaskhanian A, 2017	2007–2015	Mazandaran	Screening program	3–5	Colorimetric method and HPLC technique	193,229	214,015	407,244					Medium risk
[16]	Abbaskhanian A, 2017	2007	Mazandaran	Screening program	3–5	Colorimetric method and HPLC technique	20,759	21,569	42,328	254	2	1	1	Medium risk
[16]	Abbaskhanian A, 2017	2008	Mazandaran	Screening program	3–5	Colorimetric method and HPLC technique	22,506	23,197	45,703	66	2	0	2	Medium risk
[16]	Abbaskhanian A, 2017	2009	Mazandaran	Screening program	3–5	Colorimetric method and HPLC technique	22,050	23,392	45,442	92	7	1	6	Low risk
[16]	Abbaskhanian A, 2017	2010	Mazandaran	Screening program	3–5	Colorimetric method and HPLC technique	21,447	22,973	44,420	19	3	1	2	Medium risk
[16]	Abbaskhanian A, 2017	2011	Mazandaran	Screening program	3–5	Colorimetric method and HPLC technique	21,168	23,374	44,342	11	1	0	1	Low risk
[16]	Abbaskhanian A, 2017	2012	Mazandaran	Screening program	3–5	Colorimetric method and HPLC technique	21,719	22,633	44,352	7	2	1	1	Medium risk
[16]	Abbaskhanian A, 2017	2013	Mazandaran	Screening program	3–5	Colorimetric method and HPLC technique	22,738	23,256	45,994	5	3	0	3	Low risk
[16]	Abbaskhanian A, 2017	2014	Mazandaran	Screening program	3–5	Colorimetric method and HPLC technique	22,672	24,548	47,220	6	4	1	3	Medium risk
[16]	Abbaskhanian A, 2017	2015	Mazandaran	Screening program	3–5	Colorimetric method and HPLC technique	23,181	24,262	47,443	5	3	1	2	Low risk
[17]	Ordooei M, 2015	2010–11	Yazd	Screening program	3	Colorimetric method and HPLC technique			22,131		4	1	3	Medium risk
[18]	Motamedi N, 2017	2006	Lorestan	Screening program	Infancy	Colorimetric, ELISA and HPLC technique			33,284		4			Medium risk
[18]	Motamedi N, 2017	2007	Lorestan	Screening program	Infancy	Colorimetric, ELISA and HPLC technique			33,890		5			Medium risk
[18]	Motamedi N, 2017	2008	Lorestan	Screening program	Infancy	Colorimetric, ELISA and HPLC technique			34,045		7			Medium risk
[18]	Motamedi N, 2017	2009	Lorestan	Screening program	Infancy	Colorimetric, ELISA and HPLC technique			35,969		5			Medium risk
[18]	Motamedi N, 2017	2010	Lorestan	Screening program	Infancy	Colorimetric, ELISA and HPLC technique			35,011		6			Medium risk
[18]	Motamedi N, 2017	2011	Lorestan	Screening program	Infancy	Colorimetric, ELISA and HPLC technique			35,799		10			Medium risk
[18]	Motamedi N, 2017	2012	Lorestan	Screening program	Infancy	Colorimetric, ELISA and HPLC technique			37,154		6			Low risk
[18]	Motamedi N, 2017	2013	Lorestan	Screening program	Infancy	Colorimetric, ELISA and HPLC technique			37,944		4			Medium risk
[18]	Motamedi N, 2017	2014	Lorestan	Screening program	Infancy	Colorimetric, ELISA and HPLC technique			39,388		15			Low risk
[18]	Motamedi N, 2017	2015	Lorestan	Screening program	Infancy	Colorimetric, ELISA and HPLC technique			38,585		5			Medium risk
[18]	Motamedi N, 2017	2016	Lorestan	Screening program	Infancy	Colorimetric, ELISA and HPLC technique			36,585		7			Low risk
[19]	Ajami A, 2013	2012–13	Isfahan	Screening program	Infancy	Colorimetric method and HPLC technique			77,000		45	12	33	Medium risk
[20]	Nasiri Sh, 2013	2012–13	South Khorasan	Screening program	3	Colorimetric method and HPLC technique			26,455	31	23			Low risk
[21]	Modares Sadrani N, 2013	2012–13	Ardebil	Screening program	3	Colorimetric method and HPLC technique			44,232		13	8	5	Medium risk
[22]	Morovatdar N, 2015	2013	Razavi Khorasan	Screening program	Infancy	Colorimetric method and HPLC technique			69,347		4			Medium risk
[23]	Saadatinasab Z, 2015	2012–14	South Khorasan	Screening program	Neonatal	Colorimetric method and HPLC technique			30,103	55	3			Medium risk
[24]	Badiee M, 2014	2011–13	Torbat Heydariy	Screening program	Neonatal	Colorimetric method and HPLC technique	5390	5701	11,091		16	1	15	Medium risk
[25]	Karamifar H, 2010	2007–8	Fars	Screening program	3–5	Colorimetric method and HPLC technique	35,470	41,496	76,966	9	8	3	5	Medium risk
[26]	Soori M, 2018	2016–17	Nahavand	Screening program	Neonatal	Immuno-enzymatic method			5704	0	0	0	0	Medium risk
[27]	Heydari A, 2016	2013	All Iran	Screening program	Neonatal	Colorimetric method and HPLC technique			1,356,132		322			Medium risk
[28]	Ganji F, 2018	2012	Chaharmahal and Bakhtiari	Screening program	1–5	Colorimetric method and HPLC technique			13,022		1			Low risk
[28]	Ganji F, 2018	2013	Chaharmahal and Bakhtiari	Screening program	1–5	Colorimetric method and HPLC technique			19,612		4			Medium risk
[28]	Ganji F, 2018	2014	Chaharmahal and Bakhtiari	Screening program	1–5	Colorimetric method and HPLC technique			19,753		3			Low risk
[28]	Ganji F, 2018	2015	Chaharmahal and Bakhtiari	Screening program	1–5	Colorimetric method and HPLC technique			20,893		3			Medium risk
[29]	Mahmoodi M, 2013	2012–13	Golestan	Screening program	Neonatal	Colorimetric method and HPLC technique			74,000	32	4			Low risk
[30]	Behineh M, 2015	2007–2014	Khonj	Screening program	3–5	Fluorometric			6399		2			Medium risk
[31]	Rezabigidavarani E, 2018	2012–2016	Kerman	Screening program	At birth	Colorimetric method and HPLC technique			77,467	85	15	2	13	Low risk

^a^ hyperphenylalaninemia

^b^ Phenylketonuria

### Prevalence of suspected hyperphenylalaninemia

**Fig. 2:**
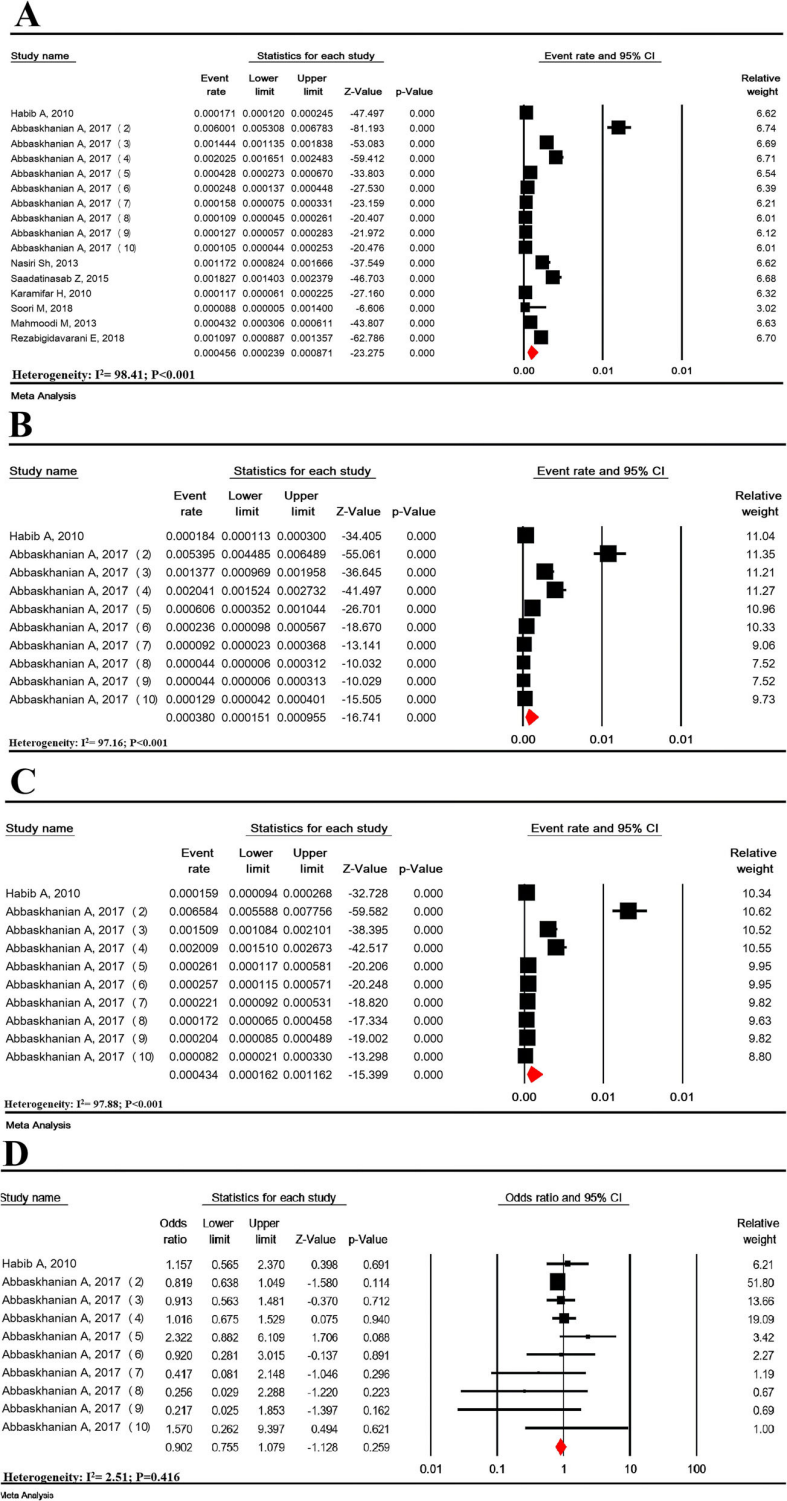
Prevalence of suspected hyperphenylalaninemia in all (**a**), girls (**b**), and boys (**c**) and girls to boys odds ratio (**d**) in national neonate screening program in Iran

Heterogeneity was high for these studies (I^2^ = 98.41%; *P* < 0.001). The prevalence of suspected HPA in 873,174 Iranian neonates was estimated to be 45.6/100,000 (95% CI: 23.9–87.1) (Fig. 2 a). The lowest prevalence was related to the study of Soori in 2016–2017 (8.8/100,000) and the highest prevalence was related to the study of Abbaskhanian in 2017 (600.1/100,000).

### Prevalence of suspected hyperphenylalaninemia based on gender

The prevalence of suspected HPA in 285,331 girls infants and 297,347 boys infants in Iran was estimated to be 38.0/100,000 (95% CI: 15.1-95.5) and 43.3/100,000 (95% CI: 16.2-116.2), respectively (Fig. 2 b-c). The girls-boys OR of suspected HPA was not significant (OR = 0.90 (95% CI: 0.75–1.08; *P* = 0.259) (Fig. 2-d).

### Subgroup analysis of the prevalence of suspected hyperphenylalaninemia

**Fig. 3:**
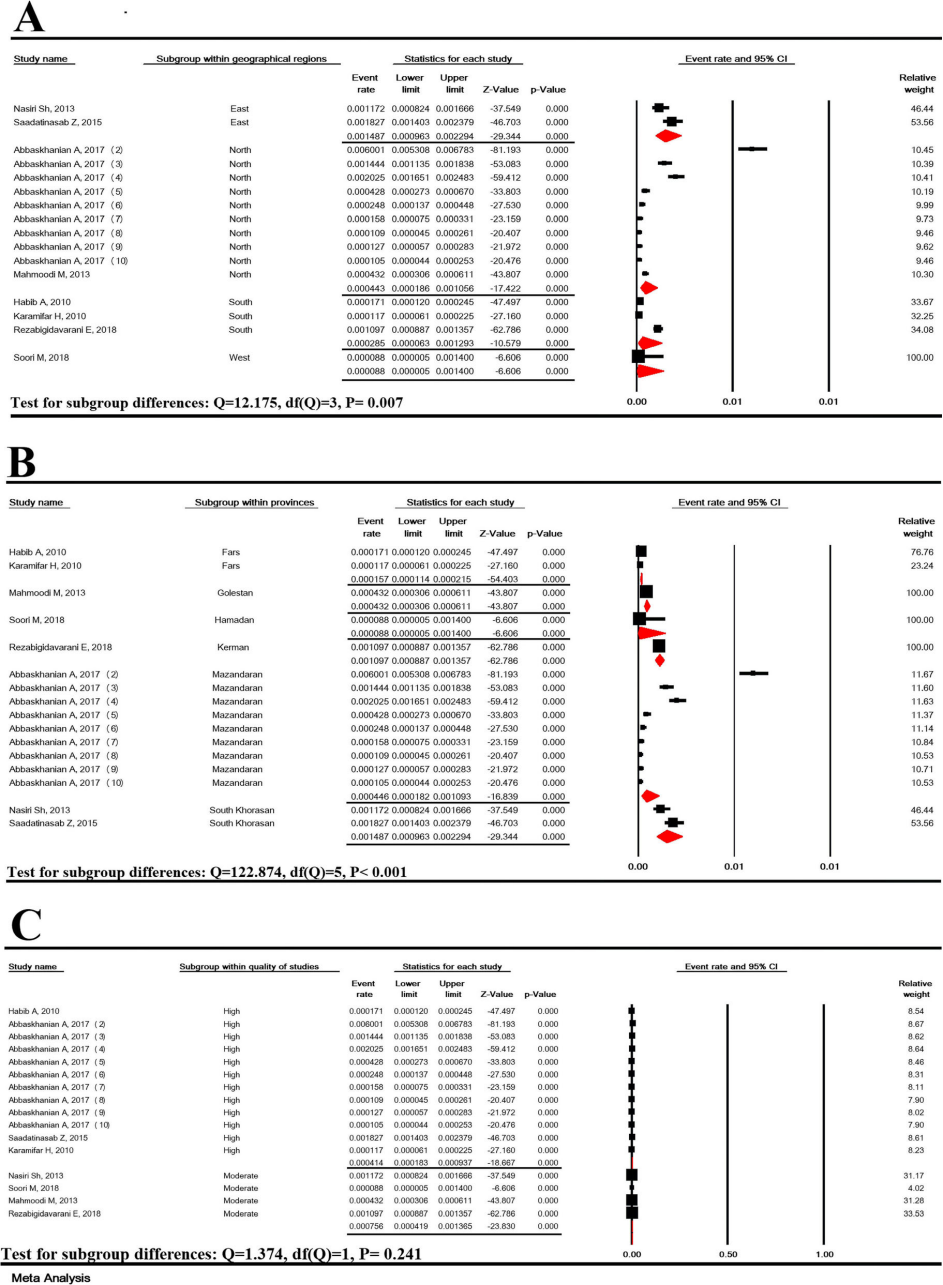
Subgroup analysis of suspected hyperphenylalaninemia prevalence based on geographical regions (**a**), provinces (**b**) and the quality of studies (**c**) in national neonate screening program in Iran

Subgroup analysis of the prevalence of suspected HPA based on five geographical regions and provinces in Iran showed significant differences with *P* < 0.007 and *P* < 0.001, respectively, but it was not significant in terms of the quality of studies (*P* = 0.241) (Fig. 3).

### Prevalence of phenylketonuria

**Fig. 4:**
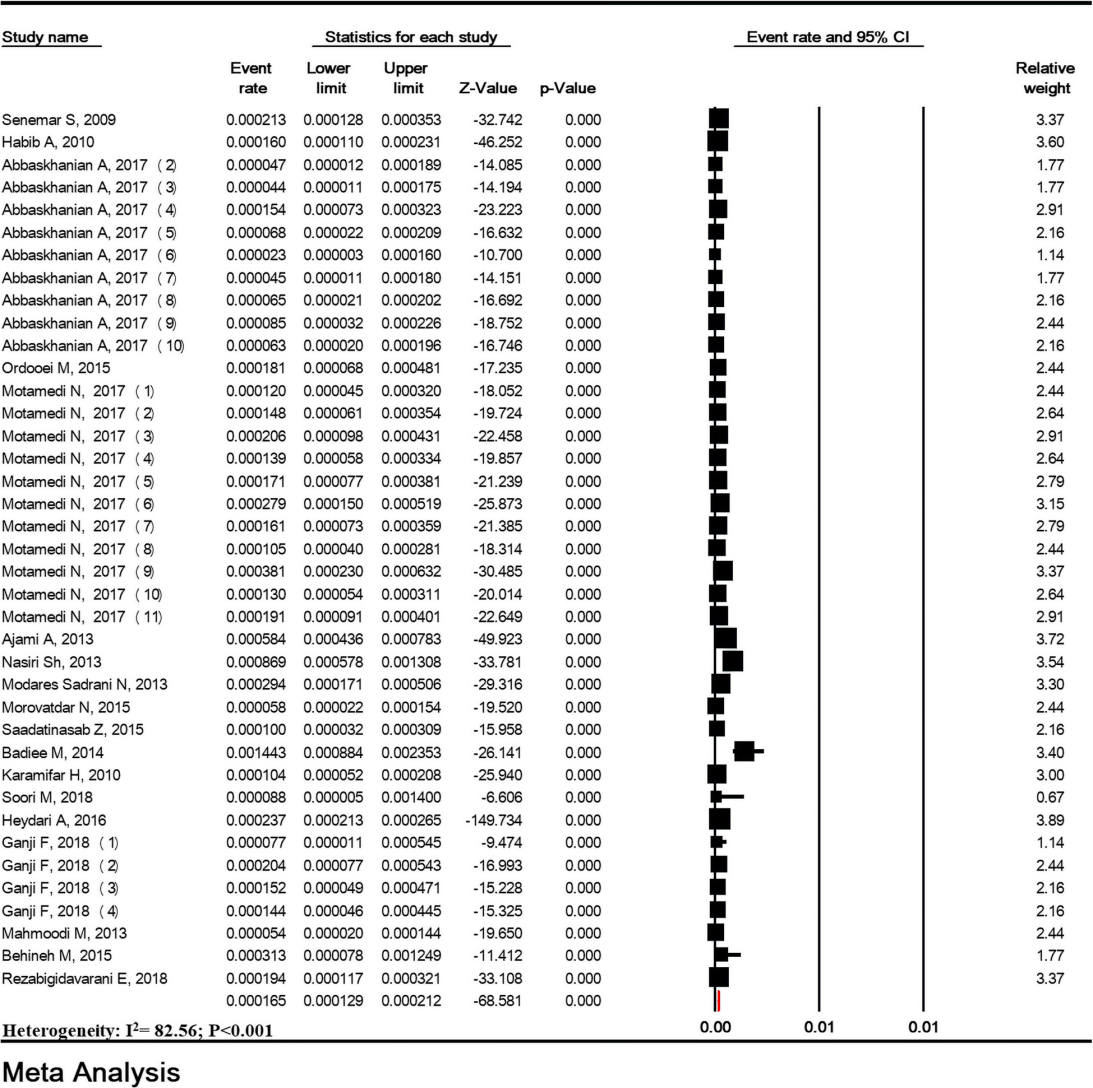
Prevalence of phenylketonuria in national neonate screening program in Iran

Heterogeneity was high for the studies (I^2^ = 82.56%; *P* < 0.001). The prevalence of PKU in 3,000,917 Iranian neonates was estimated to be 16.5/100,000 (95% CI: 12.9–21.2). The lowest and highest prevalence was related to the studies of Abbaskhanian et al. (2.3/100,000) and Badiee et al. (144.3/100,000) (Fig. 4), respectively.

### Prevalence of phenylketonuria based on gender

**Fig. 5:**
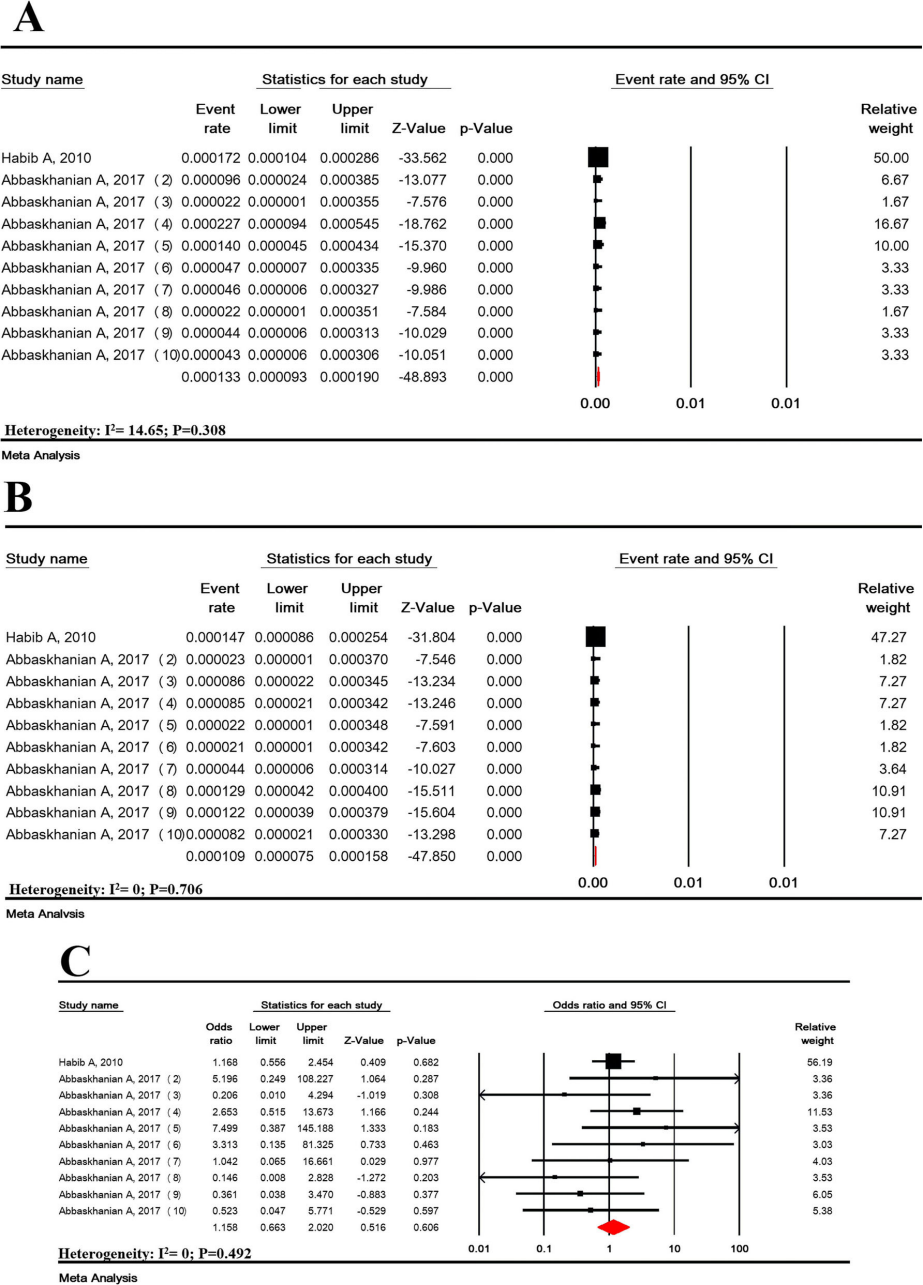
Prevalence of phenylketonuria in girls (**a**) and boys (**b**) and girls to boys odds ratio (**d**) in national neonate screening program in Iran in Iran

The prevalence of PKU in 285,331 girls and 297,347 boys infants was estimated to be 13.3/100,000 (95% CI: 9.3-19.0) and 10.9/100,000 (95% CI: 7.5–15.8), respectively. The girls-boys OR of PKU prevalence was not significant (OR = 1.58 (95% CI: 0.66–2.02, *P* = 0.606) (Fig. 5).

### Subgroup analysis of phenylketonuria prevalence

The prevalence of PKU in West, East, North, South and Center of Iran was estimated to be 19.4/100,000 (95% CI: 15.1–24.9), 31.9/100,000 (95% CI: 9.0–113.3), 7.9/100,000 (95% CI: 4.7–13.1), 17.2/100,000 (95% CI: 13.6–21.8) and 21.3/100,000 (95% CI: 10.2–44.6), respectively, and the differences in subgroup analysis were significant (*P* < 0.001). In subgroup analysis based on province, the lowest and highest prevalence of PKU was in Golestan (5.4/100,000) and Isfahan (58.4/100,000) provinces, respectively and the difference was significant (*P* < 0.001).

**Fig. 6:**
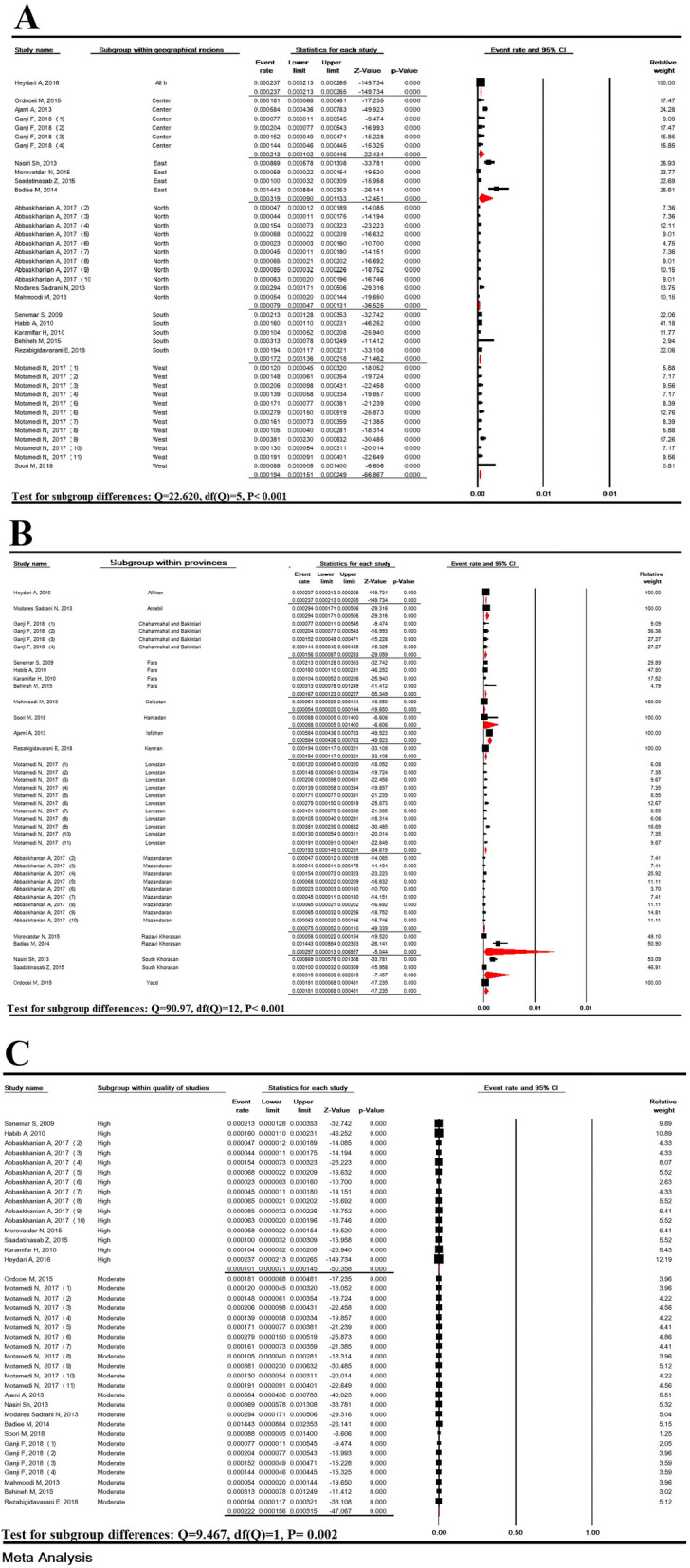
Subgroup analysis of phenylketonuria prevalence based on geographical regions (**a**), provinces (**b**) and the quality of studies (**c**) in national neonate screening program in Iran

In subgroup analysis based on the quality of studies, the prevalence of PKU in medium and high-quality studies was estimated to be 22.2/100,000 (95% CI: 15.6-31.5) and 10.1/100,000 (95% CI: 7.1-14.5), respectively, and the differences in subgroup analysis were significant (*P* = 0.002) (Fig. 6).

### The prevalence of different types of phenylketonuria

**Fig. 7:**
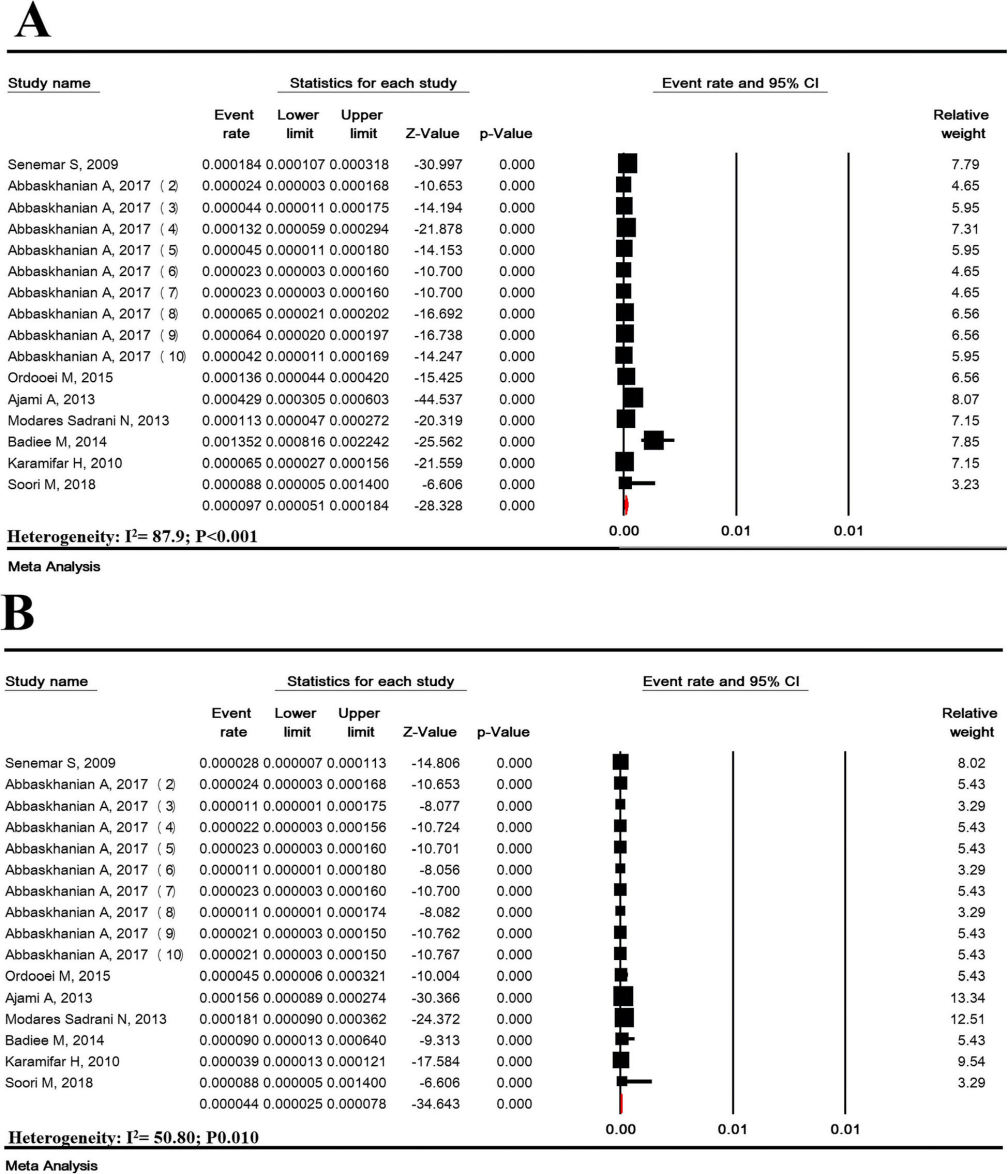
Prevalence of mild to moderate hyperphenylalaninemia (**a**) classic phenylketonuria (**b**) in national neonate screening program in Iran

The prevalence of PKU types was investigated in 16 studies with a sample size of 714,845 Iranian neonates. The prevalence of mild to moderate HPA was estimated 9.7/100,000 (95% CI: 5.1–18.4) and the prevalence of classical PKU was estimated 4.4/100,000 (95% CI: 2.5–7.8) (Fig. 7). In other words, the prevalence of mild to moderate HPA and the prevalence of classical PKU among PKU patients were 71.15% (95% CI: 61.88–78.93) and 28.85% (95% CI: 21.07–38.12), respectively (Supplementary Figure 1).

### Meta-regression

Meta-regression for the prevalence of suspected HPA, PKU, classical PKU and mild to moderate HPA based on the year of study was (meta-regression coefficient: - 0.132, 95% CI − 0.346 to 0.081, *P* = 0.226), (meta-regression coefficient: 0.008, 95% CI − 0.076 to 0.92, *P* = 0.851), (meta-regression coefficient: 0.101, 95% CI − 0.213 to 0.416, *P* = 0.528) and (meta-regression coefficient: 0.020, 95% CI − 0.212 to 0.416, *P* = 0.253), respectively (Supplementary Figure 2).

### Sensitivity analysis and publication bias

Sensitivity analysis for prevalence of suspected HPA, PKU, classical PKU, and mild to moderate HPA with the omission of one study showed that overall estimation is still robust (Supplementary Figures 3, 4, 5). Egger’s and Begg’s tests for HPA prevalence (*P* = 0.137 and *P* < 0.001, respectively), overall PKU prevalence (*P* = 0.005 and *P* = 0.009), classical PKU (*P* < 0.001 and *P* = 0.002) and mild to moderate PKU (*P* = 0.710 and *P* < 0.001) were significant (Supplementary Figure 6).

## Discussion

The present study is the first comprehensive systematic review and meta-analysis on PKU screening in Iranian newborns. The prevalence of PKU in Iranian neonates was estimated to be 16.5/100,000. NBS is an important form of prevention in newborns with congenital metabolic diseases. This activity is very useful for detecting errors in many birth-related errors. It should be noted that many types of congenital disorders can be successfully treated if early diagnosis is achieved. If specific biochemical supplements are taken at an early stage, many metabolic disorders will be stopped from progressing and causing permanent damage to patients. The NBS is managed under the national public health policies. Metabolic disorders of the nervous system vary clinically and pathologically. Mental retardation and epilepsy syndrome are prominent in these disorders [42, 43].

The first pilot study to assess HPA in neonates in Iran began in 1982 [44] and the first National Neonate Screening Program (NNSP) in Iran started in Fars province in 2004 [45] and continued in Mazandaran province in 2007 [16]. Based on law, all Iranian infants should be screened for three diseases, including hypothyroidism, PKU, and glucose 6-phosphate dehydrogenase deficiency.

The published evidence confirms that the global NBS for PKU meets all accepted screening criteria and justifies the cost and infrastructure needed to collect and test dried blood spots [46]. Screening in infants is considered a national duty even in countries where there is no PKU population. Because of high migration in countries, detection of PKU has remained probable. Neonatal screening requires: 1) a solid infrastructure in which blood is collected from all neonates to ensure timely treatment; 2) a well-equipped laboratory that can effectively administer blood spot. Low-income countries may consider using NBS laboratory facilities of other countries [47, 48].

In the present study, the prevalence of suspected HPA (45.6/100,000) was much higher than the prevalence of PKU. Differential diagnosis of HPA includes high intake of natural protein, liver disease, tetrahydrobiopterin (BH4) deficiency, and being premature [49, 50].

The prevalence of PKU varies across ethnic groups and geographical regions around the world [51]. The prevalence of PKU has been reported to range from less than 1/220,000 to 1/4500. Table 2 summarizes the prevalence of PKU in different populations. In the present study, due to ethnic differences in different geographical regions of Iran, the prevalence of PKU was significantly different in five different regions of Iran.

**Table 2:** Summarizes the prevalence of PKU in different populations

Regions	Countries	Incidence of PKU^a^
Asian populations	Turkey, 1986 (53)	1: 4500
	Saudi Arabia, 2017 [8]	1: 14245
	United Arab of Emirates, 2000 (54)	1: 20050
	United Arab of Emirates, 2014 (55)	1: 14544
	Iraq, 2015 (56)	1.2: 10000
	Thailand 2009 and 2015 (13, 57)	< 1: 220,000
	Mexico, 2012 (58)	1:161,748
	Japan, 2017 (59)	1:143,000
European populations	Ireland, 1978 (60)	1 in 4500
	Sweden, before and after 1990 [52]	1:18,300 to 1:14,200
	Germany, 2002 and 2014 (61)	1:10,339
	Greece, 2016 (62)	1: 10000
	Bulgaria, 2016 (62)	1: 18000
	Poland, 2016 (62)	1: 7000
	Spain, 2016 (62)	1: 7400
	Italy, 2016 (62)	1: 11500
South America	Brazil, 2014 (63)	1:8690
North America	United States (Caucasians) (64)	1 in 10,000
	Canada, 1986 (65)	1 in 22,000

^a^ Phenylketonuria

In the present study, prevalence of mild to moderate HPA and prevalence of classical PKU among PKU patients were 71.15 and 28.85%, respectively, indicating that the majority of patients with PKU suffer from mild to moderate HPA in Iran. This can play a key role in the initiation and non-initiation of PKU treatment and management of these patients. That’s because untreated Phe concentration determines the management of people with PKU. If blood Phe concentrations are below 360 μmol/l, no intervention is required. If Phe blood concentration is between 360 μmol/l and 600 μmol/l, treatment up to the age of 12 is recommended and lifelong treatment is recommended if the concentration is above 600 μmol/l. For women trying to get pregnant (maternal PKU), untreated Phe blood concentration drops to more than 360 μmol/l. On the other hand, management of PKU is associated with a severe financial burden on patients’ families, which may lead to inadequate treatment or a change in blood Phe concentration [2, 3].

The trend of changes in the prevalence of suspected HPA, PKU, classical PKU and mild to moderate HPA did not change significantly over time. PKU as an autosomal-recessive disorder is not only related to consanguineous marriage and also occurs in regions with a low incidence of consanguineous marriage e.g. Europe [52]. In Iran, three main areas of prevention and control of hereditary metabolic diseases for PKU include NSB for this disease, selected one-stop clinics and pre-marriage screening program. Therefore, one can say that Iran’s national programs regarding pre-marriage genetic counseling have not been effective in reducing PKU.

This study has several strengths: 1) A comprehensive search strategy was used in this study to maximize the possibility of identifying all relevant literature and even gray literature; 2) All research steps were conducted independently by two researchers, and disagreements were resolved by group discussion, 3) To obtain additional information and to make decisions about duplicate publication, we contacted the authors of the studies, 4) In cases where heterogeneity was significant, the random effects model was used to integrate data to provide a conservative estimate and on the other hand, subgroup analysis and meta-regression model were used to find the cause of heterogeneity and publication bias, and 5) We excluded studies on certain patients such as mentally retarded patients or studies with non-random sample and the resulting estimate can be generalized to the total population.

The limitations of the present study include the limitation of Iranian databases in combined search. In addition, there was a high heterogeneity among studies that investigated the prevalence of suspected HPA and PKU, and based on the available data, we were only able to do subgroup analysis based on geographical regions and provinces, which was significant. Therefore, the differences between studies can be attributed to these issues. However, it seems that more important issues such as differences in the percentage of consanguineous marriages and genetic differences between different populations of Iran (given that Iran includes various ethnic groups) may also be the reason for differences between studies. It was not possible to address these issues in this study. Other studies including the study of Hardelid et al. in England showed that the prevalence of PKU is lower among the Sub-Saharan Africans and South Asians who migrated to England [51]. Studies in other European countries showed that the increased incidence of PKU may be due to new mutations in this disease and migration of people of different races to their country [52].

## Conclusions

The prevalence of PKU in Iran was estimated to be 16.6/100,000 or 1/6.024. Due to ethnic and demographic similarities in Iran, we may also expand our results and estimates to Iranians living in other countries. It should be considered that for phenylketonuria there is a highly effective dietary treatment which can prevent the clinical symptoms of phenylketonuria if initiated early after detection by newborn screening.

## Supplementary information

**Additional file 1: Figure 1.** Prevalence of mild to moderate hyperphenylalaninemia (**a**) and classic phenylketonuria (**b**) among phenylketonuria patients.

**Additional file 2: Figure 2.** Meta-regression model for prevalence of suspected hyperphenylalaninemia (**a**), phenylketonuria (**b**), classic phenylketonuria (**c**), and mild to moderate hyperphenylalaninemia (**d**) based on year of study.

**Additional file 3: Figure 3.** Sensitivity analysis for prevalence of suspected hyperphenylalaninemia in national neonate screening program in Iran.

**Additional file 4: Figure 4.** Sensitivity analysis for prevalence of phenylketonuria in national neonate screening program in Iran.

**Additional file 5: Figure 5.** Sensitivity analysis for prevalence of mild to moderate hyperphenylalaninemia (**a**) classic phenylketonuria (**b**) in national neonate screening program in Iran.

**Additional file 6: Figure 6.** Publication bias for prevalence of suspected hyperphenylalaninemia (a), phenylketonuria (**b**), classic phenylketonuria (**c**), and mild to moderate hyperphenylalaninemia (**d**) based on year of study.

## Data Availability

Not applicable.

## Abbreviations

PKU: Phenylketonuria
PAH: Phenylalanine hydroxylase
Phe: Phenylalanine
HPA: Hyperphenylalaninemia
NBS: Newborn screening
RICST: Regional Information Center for Science and Technology
SID: Scientific Information Database
IranDoc: Iranian Research Institute for Information Science and Technology
NNSP: National Neonate Screening Program
MOOSE: Meta-analyses Of Observational Studies in Epidemiology
PRISMA: Systematic Reviews and Meta-analysis
OR: Odds ratio
CI: Confidence interval
DBS: Dried Blood Spot
HPLC: high-Performance Liquid Chromatographic
PROSPERO: International prospective register of systematic reviews
PICO: Patient, Population, or Problem; Intervention, Prognostic Factor, or Exposure; Comparison or Intervention (if appropriate); Outcome
NOS: Newcastle-Ottawa Scale
BH4: Tetrahydrobiopterin
