# Supplementary figures and images for: Phenylketonuria screening in Iranian newborns: a systematic review and meta-analysis
Source: BMC Pediatr. 2020 Jul 24;20:352. doi: 10.1186/s12887-020-02230-6 (PMC7379797; doi:10.1186/s12887-020-02230-6)

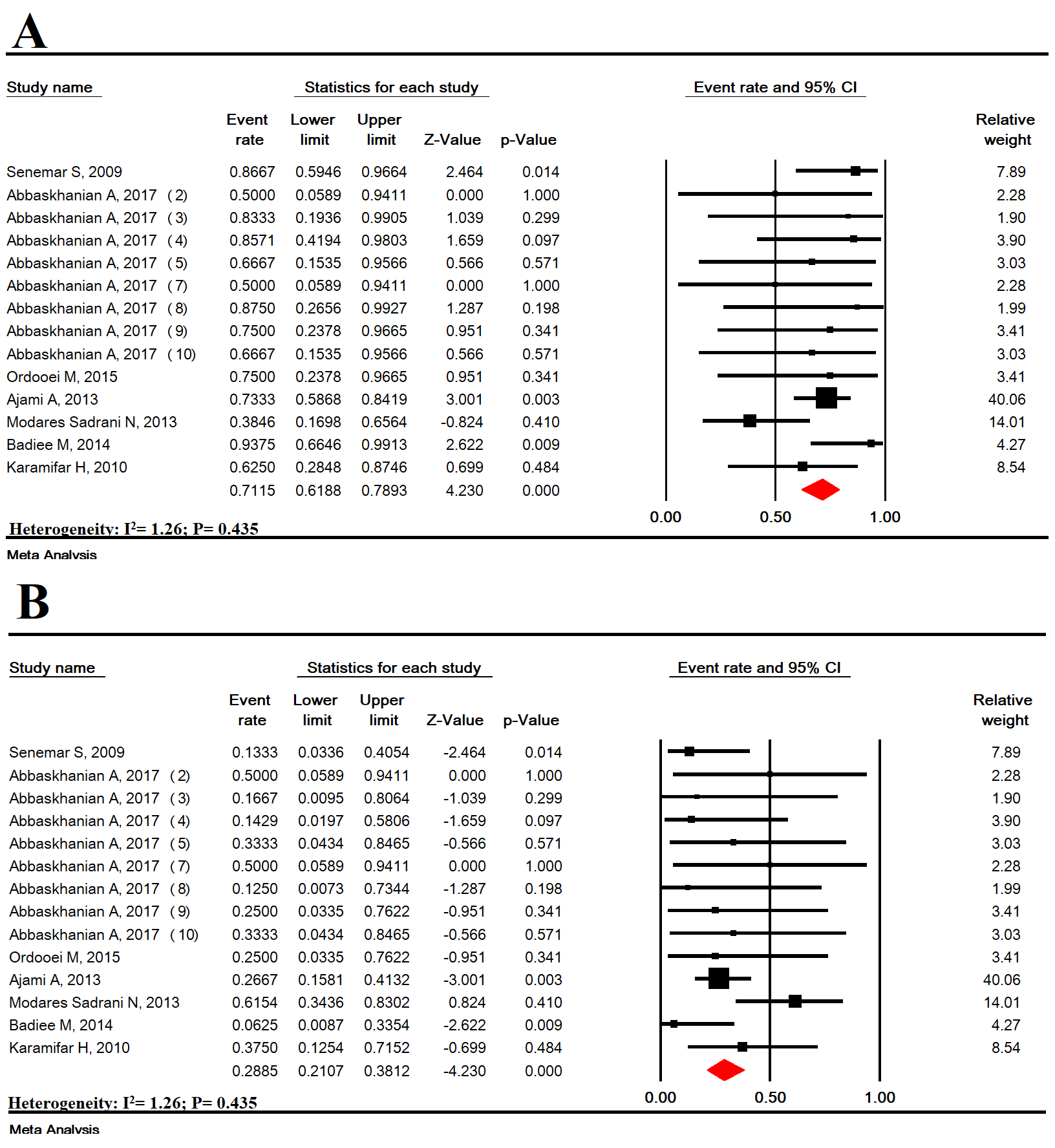

Supplement: Supplementary file 1 — Additional file 1: Figure 1. Prevalence of mild to moderate hyperphenylalaninemia (a) and classic phenylketonuria (b) among phenylketonuria patients. [file 12887_2020_2230_MOESM1_ESM.tif]

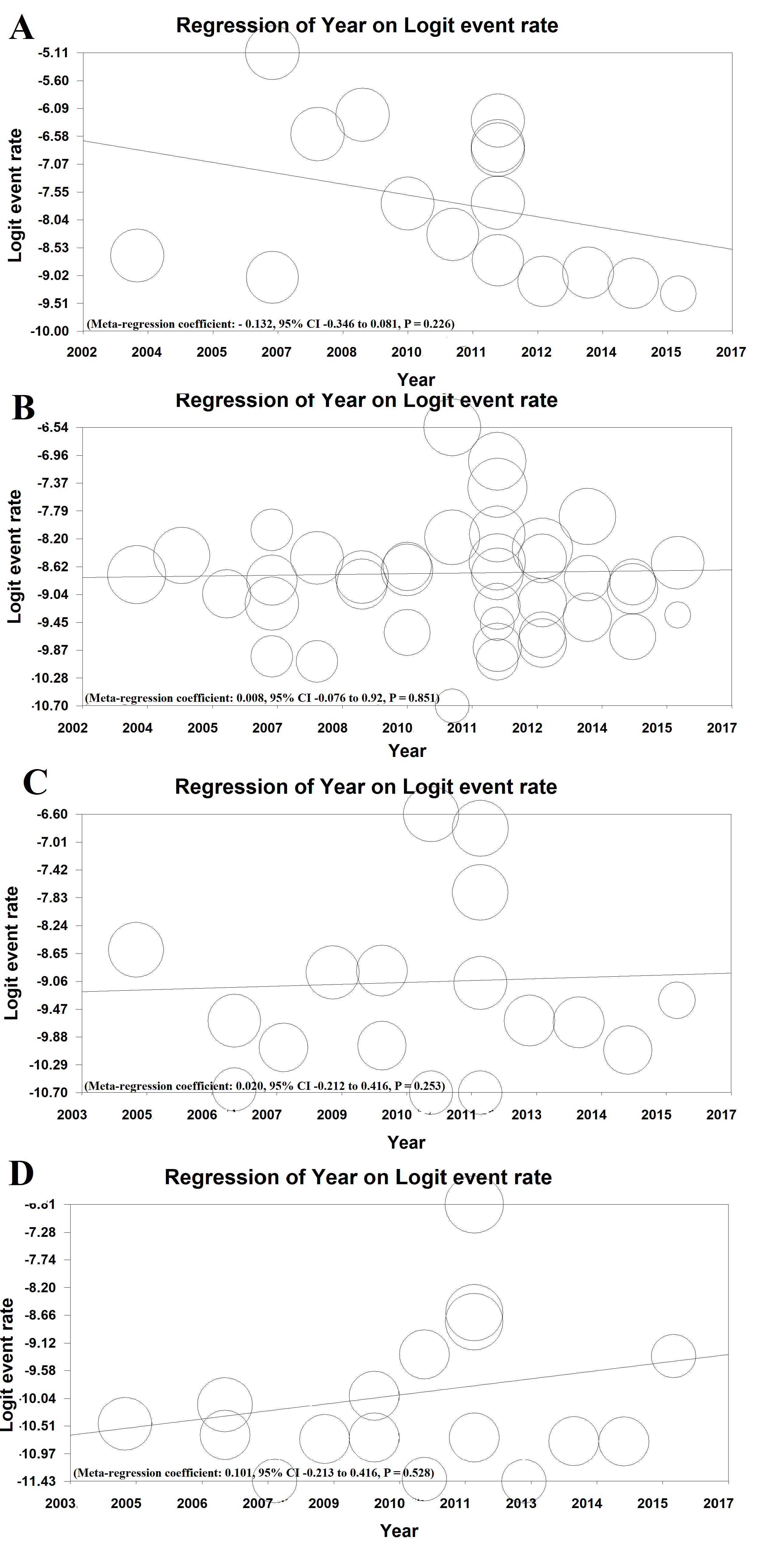

Supplement: Supplementary file 2 — Additional file 2: Figure 2. Meta-regression model for prevalence of suspected hyperphenylalaninemia (a), phenylketonuria (b), classic phenylketonuria (c), and mild to moderate hyperphenylalaninemia (d) based on year of study. [file 12887_2020_2230_MOESM2_ESM.tif]

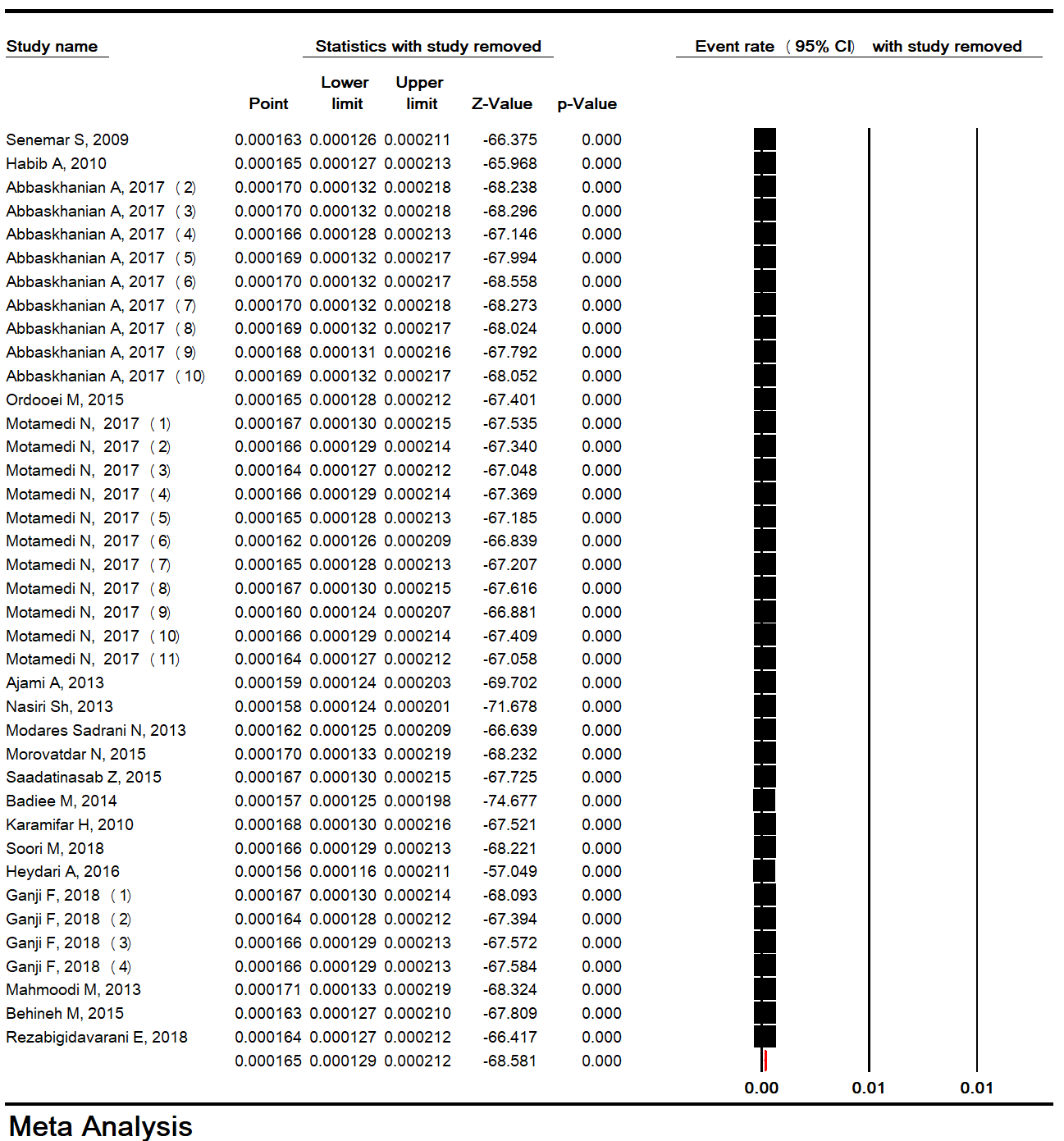

Supplement: Supplementary file 3 — Additional file 3: Figure 3. Sensitivity analysis for prevalence of suspected hyperphenylalaninemia in national neonate screening program in Iran. [file 12887_2020_2230_MOESM3_ESM.tif]

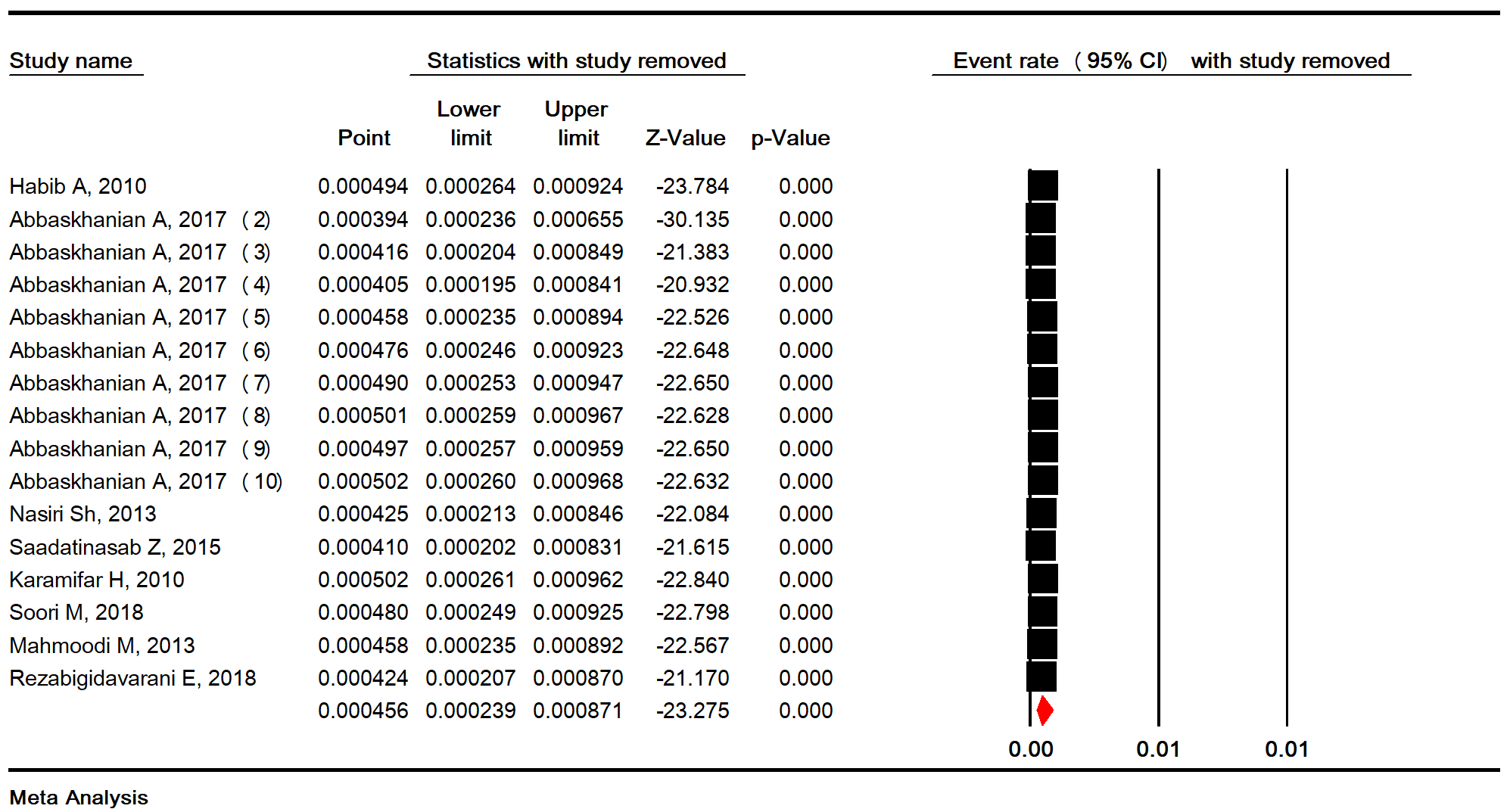

Supplement: Supplementary file 4 — Additional file 4: Figure 4. Sensitivity analysis for prevalence of phenylketonuria in national neonate screening program in Iran. [file 12887_2020_2230_MOESM4_ESM.tif]

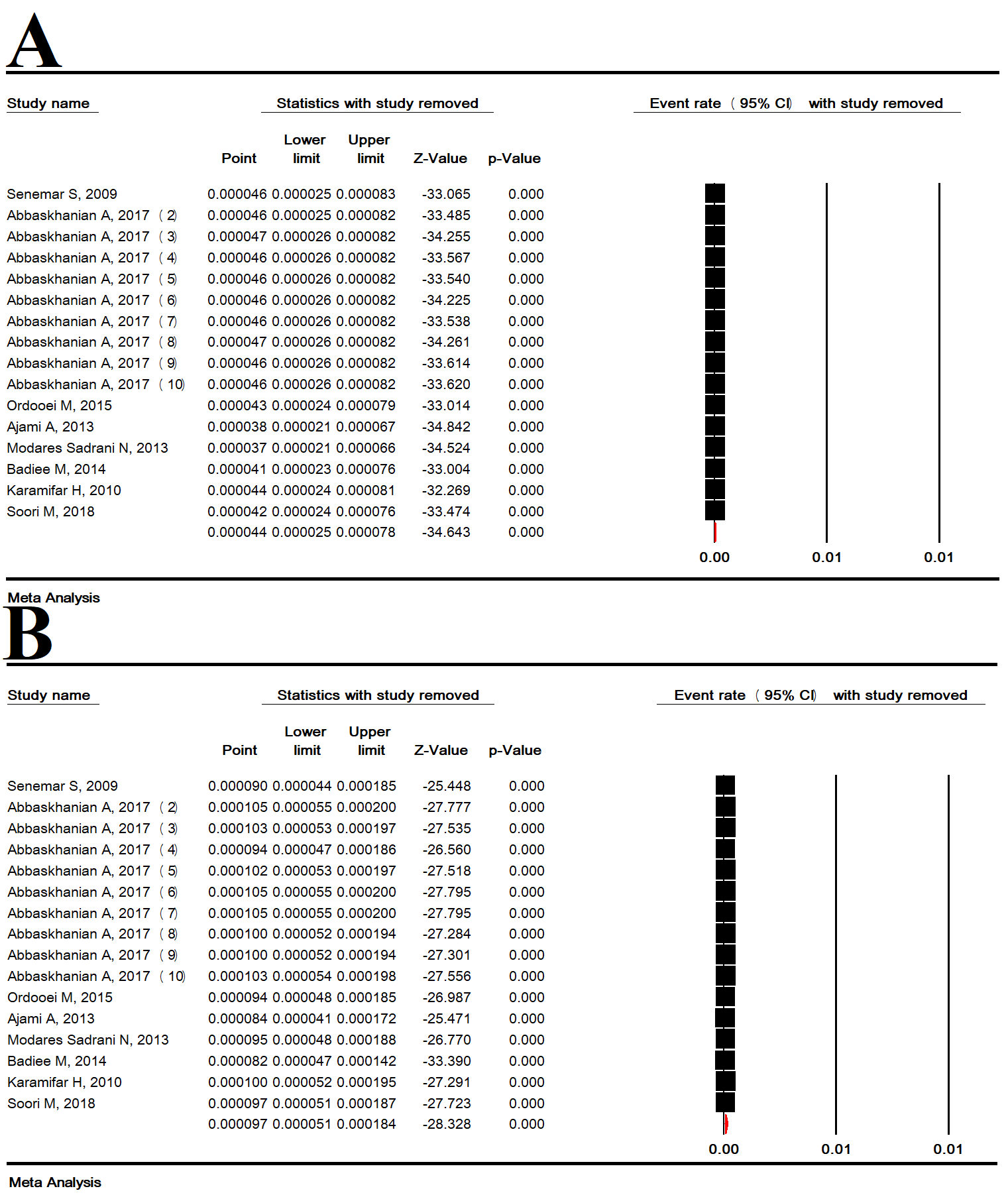

Supplement: Supplementary file 5 — Additional file 5: Figure 5. Sensitivity analysis for prevalence of mild to moderate hyperphenylalaninemia (a) classic phenylketonuria (b) in national neonate screening program in Iran. [file 12887_2020_2230_MOESM5_ESM.tif]

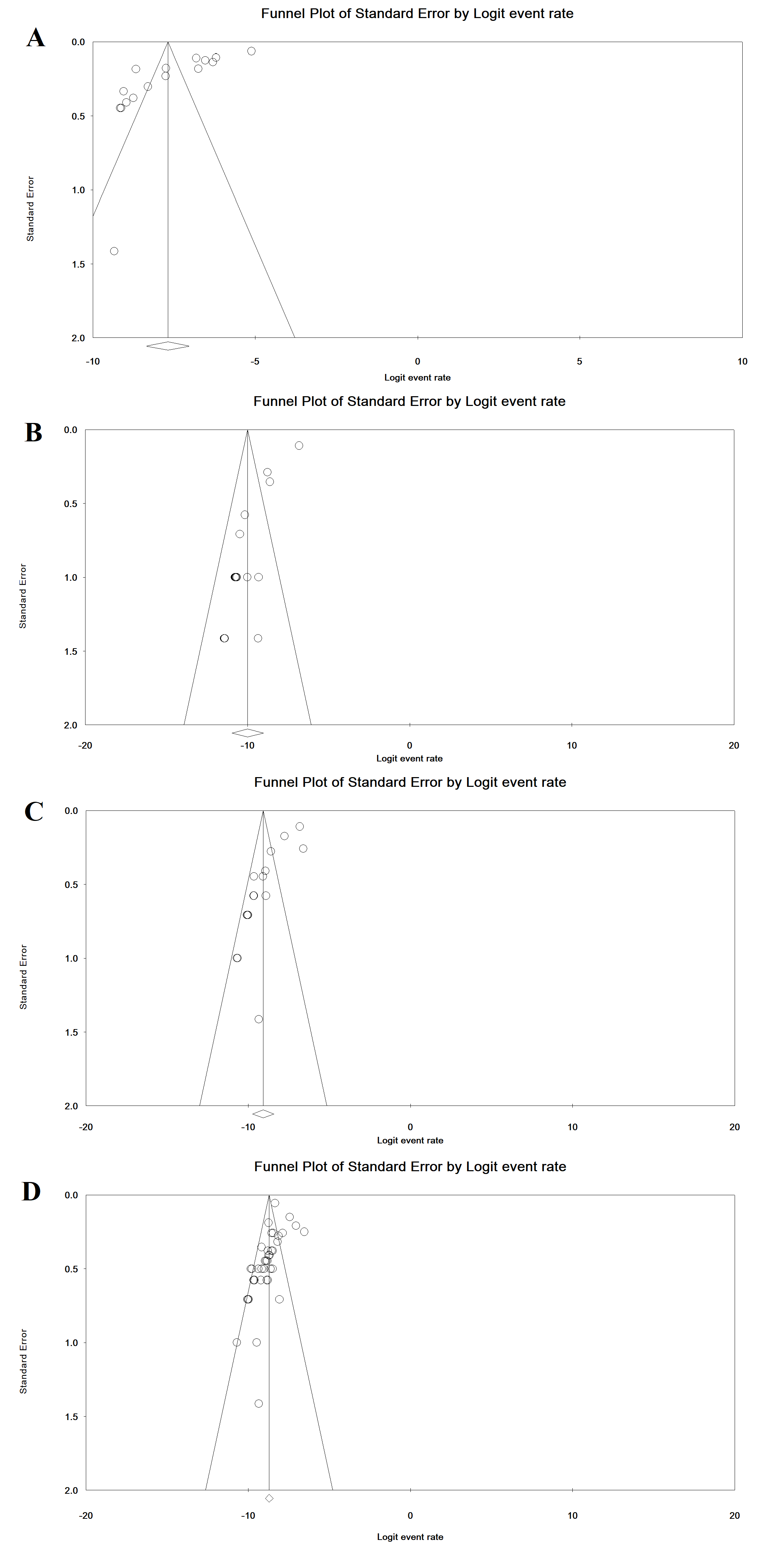

Supplement: Supplementary file 6 — Additional file 6: Figure 6. Publication bias for prevalence of suspected hyperphenylalaninemia (a), phenylketonuria (b), classic phenylketonuria (c), and mild to moderate hyperphenylalaninemia (d) based on year of study. [file 12887_2020_2230_MOESM6_ESM.tif]
